# Streamlining Decision Making in Contralateral Risk-Reducing Mastectomy: Impact of PREDICT and BOADICEA Computations

**DOI:** 10.1245/s10434-018-6593-4

**Published:** 2018-07-17

**Authors:** Tania Samantha de Silva, Victoria Rose Russell, Francis Patrick Henry, Paul Thomas Ryan Thiruchelvam, Dimitri John Hadjiminas, Ragheed Al-Mufti, Roselyn Katy Hogben, Judith Hunter, Simon Wood, Navid Jallali, Daniel Richard Leff

**Affiliations:** 1 Breast Unit, Imperial College Healthcare NHS Trust, London, UK; 2 Plastics and Reconstructive Surgery, Imperial College Healthcare NHS Trust, London, UK; 3 Department of Surgery and Cancer, BioSurgery and Surgical Technology, 10th Floor, QEQM Wing, St Mary’s Hospital, Paddington, London, W2 1NY UK

## Abstract

**Introduction:**

Patients with sporadic breast cancer (BC) have low contralateral breast cancer risk (CLBCR; approximately 0.7% per annum) and contralateral prophylactic mastectomy (CPM) offers no survival advantage. CPM with autologous reconstruction (AR) has major morbidity and resource implications.

**Objective:**

The aim of this study was to review the impact of PREDICT survival estimates and lifetime CLBCR scores on decision making for CPM in patients with unilateral BC.

**Methods:**

Of *n* = 272 consecutive patients undergoing mastectomy and AR, 252 were included. Five- and 10-year survival was computed with the PREDICT(V2) online prognostication tool, using age and clinicopathological factors. Based on family history (FH) and tumor biology, CLBCR was calculated using validated BODICEA web-based software. Survival scores were correlated against CLBCR estimates to identify patients receiving CPM with ‘low’ CLBCR (< 30% lifetime risk) and poor prognosis (5-year survival < 80%). Patients with ‘high’ CLBCR receiving unilateral mastectomy (UM) were similarly identified (UK National Institute of Health and Care Excellence [NICE] criteria for CPM, ≥ 30% lifetime BC risk). Justifications motivating CPM were investigated.

**Results:**

Of 252 patients, 215 had UM and 37 had bilateral mastectomy and AR. Only 23 (62%) patients receiving CPM fulfilled the NICE criteria. Of 215 patients, 5 (2.3%) failed to undergo CPM despite high CLBCR and good prognosis. CPMs were performed, at the patient’s request, for no clear justification (*n* = 8), contralateral non-invasive disease, and/or FH (*n* = 5), FH alone (*n* = 4) and ipsilateral cancer recurrence-related anxiety (*n* = 3).

**Conclusion:**

In the absence of prospective risk estimates of CLBCR and prognosis, certain patients receive CPM and reconstruction despite modest CLBCR, yet a proportion of patients with good prognoses and substantial risk are not undergoing CPM.

Several factors, including genetics, environment and lifestyle, contribute to breast cancer (BC) risk.^1^ For patients with sporadic unilateral disease, the risk of contralateral breast cancer (CLBC) is 0.1–0.6% per annum,^2^,^3^ whereas BRCA carriers have a 30–40% actual risk at 15 years.^4^ Contralateral prophylactic mastectomy (CPM) in patients with unilateral BC only confers significant risk reduction in patients with high-risk family or genetic mutations but no survival advantage.^5^ On these grounds, the UK National Institute of Health and Care Excellence (NICE) recommended CPM for predisposing genetic mutations and CLBC risk > 30%.^6^ CPM is not recommended if life expectancy is limited or comorbidities increase perioperative risks.^6^ In 2016, the American Society of Breast Surgeons consensus statement advised CPM on the grounds of strong family history (FH) of BC, genetic mutations, and mantle chest radiotherapy before age 30 years.^7^

Literature review depicts declining CLBC incidence, attributed to advances in BC treatment and hormonal therapy.^2^,^3^ Moreover, risk of death from index cancer metastasis is greater than the risk of death from CLBC.^8^,^9^ Despite this, CPM has more than doubled in the UK and risen by 150% in the US,^5^,^9^,^10^ The reasons are multifactorial and include perceived high-risk of CLBC,^11^ increased genetic testing,^12^ magnetic resonance imaging (MRI) scanning,^13^,^14^ desire for reconstructive symmetry, and anxiety.^15^ Indeed, patients’ perceived risk versus actuarial contralateral breast cancer risk (CLBCR) is often inflated, and skewed perceptions may drive CPM.^11^ Our anecdotal experience is that CPM is driven by the patient, and not entirely on the grounds of balancing CLBCR against survival from the index disease. Similarly, there is an association between CPM and reconstructive surgery, indicating restoring cosmesis and decreasing perceived CLBCR provides an incentive for CPM;^16^–^18^ however, these perceived benefits must be traded off against harms of surgery.

CPM has substantial cost implications, physical morbidity,^5^,^19^,^20^ and psychological impact with negative body image.^8^,^21^ Moreover, resource implications associated with CPM include theatre time, hospital stay, surgeons’ time, histological analysis, and secondary reconstructive procedures.^8^ CPM doubles the risk of complications, with unanticipated reoperation rates of 4% in simple CPM, versus 49% when coupled with reconstruction.^5^ If adjuvant therapy is delayed, this may impact on prognosis.^19^,^20^ Therefore, it is imperative that patients carefully weigh decisions, ensuring CPM is offered to those who benefit maximally from risk reduction.

We observed an increase in mastectomy and autologous breast reconstruction (ABR) rates at our institution since 2013 (Fig. 1). While all ABR requests are discussed in a multidisciplinary meeting, there is no contemporaneous CLBC score or survival estimates from index surgery to guide decision making. In this context, the potential risk is that certain patients with low CLBCR and/or unfavorable prognosis from their index disease may receive CPM, while those whose lifetime risk is elevated due to strong FH may not. This is critically important since patients undergoing unilateral deep inferior epigastric perforator (DIEP) flap reconstruction cannot undergo the procedure again, and hence staged CPM in high-risk patients has reconstructive consequences. Similarly, for low-risk patients, undergoing bilateral surgery exposes them to all the potential risks of complex surgery,^5^,^19^,^20^ without significant risk reduction or improved survival.^5^ Finally, prognosis from index BC may preclude CPM in certain patients with poor prognosis since they may not live long enough to see the contralateral disease. Given these factors, the objective was to evaluate indications for CPM in patients undergoing ABR at a London Oncoplastic Centre. We sought to determine the impact of estimates of CLBCR and survival on decision making for CPM, and establish whether these estimates better identify patients with high CLBCR and good prognosis to derive maximal benefit from CPM.

**Fig. 1:**
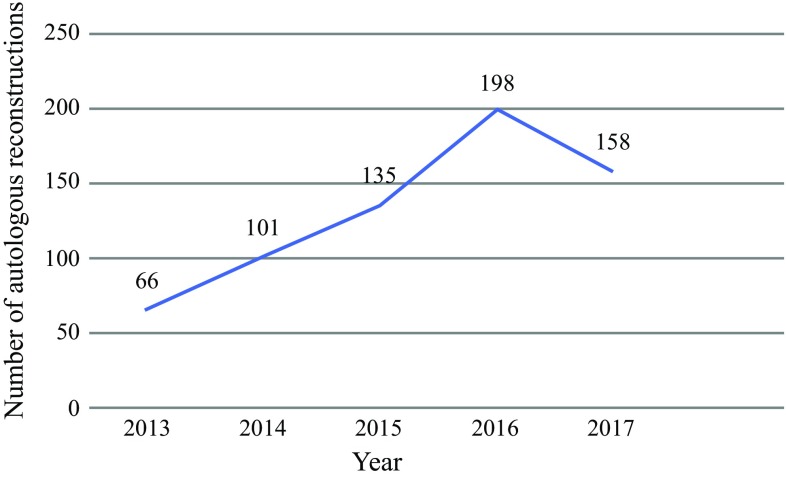
Trends in autologous reconstruction at Charing Cross Hospital between 2013 and 2017

## Patients and Methods

### Patient Identification, Clinical and Demographic Data

A retrospective analysis was conducted of all patients receiving mastectomy and ABR for BC between 2 January 2013 and 23 December 2015 at Imperial College Healthcare NHS Trust. The Institutional Review Board for Audit and Service Evaluation approved the study (registration ID 168). Two reviewers (TdS and VR) extracted the following data from a prospective ABR database: patient demographic and clinicopathological data and treatment characteristics, FH, results of genetic testing, type of surgery, duration of procedure, length of inpatient stay, and postoperative complications. Information regarding FH and reasons for CPM were collected prospectively from phone calls to individual patients. CPM was defined as the removal of a healthy breast, either simultaneous with BC treatment or subsequently.^22^

### PREDICT

PREDICT (V2) is a validated online BC prognostication and treatment response prediction model endorsed by the American Joint Committee on Cancer.^23^ In this study, the PREDICT web-based application was used to calculate 5- and 10-year survival. Clinicopathological data, such as tumor grade, tumor size, number of involved lymph nodes, and receptor status, enabled survival estimates to be computed. Modified PREDICT (v2) scores were calculated based on actual treatment(s) received. For example, if a patients’ 10-year survival without adjuvant treatment was 48.1%, and additional benefit from endocrine treatment was 12.2%, 18% from chemotherapy, and 2.2% from trastuzumab, then for a patient who received all three treatments, the modified 10-year survival was calculated to be 91.6%.

### BODCIEA

Breast and Ovarian Analysis of Disease Incidence and Carrier Estimation Algorithm (BOADICEA) BWA V3 is an externally validated risk prediction model.^24^ This web-based software tool uses tumor characteristics (age, estrogen receptor, progesterone receptor, HER2, and cyclin-dependent kinase), FH of breast, ovarian and prostate cancer (including age at diagnosis and death), known genetic mutations, and Ashkenazi Jewish ancestry to build pedigrees beyond the second-generation.^25^ BODICEA computes the probabilities of harboring BRCA1 and BRCA2 gene mutations, and estimates age-specific risks of BC until 80 years.^26^ BOADICEA accurately predicts observed mutations.^27^

### Postoperative Complications

Prospective records were reviewed to capture operating times (minutes) from the SurgiNet^®^–Cerner platform, and postoperative complications (classified as per the Clavien–Dindo classification,^28^ and length of hospital stay (days) from Cerner Millennium^®^ electronic patient records.

### Statistical Methods

Categorical outcome variables, including genetic mutation status and complications, were expressed as proportions or percentages, and continuous outcomes, such as 5- and 10-year survival estimates (PREDICT), CLBCR (BOADICEA), and theatre time (minutes), were expressed as mean (standard deviation [SD]). Statistical analysis was performed using SPSS version 20 (IBM Corporation, Armonk, NY, USA), with the significance threshold set at α = 5%. Comparisons were made between patients undergoing unilateral mastectomy (UM) versus bilateral mastectomy (BM) [with CPM]. For categorical outcome data, these comparisons were computed using Fisher’s exact test. For continuous data, such as age, 5- and 10-year survival estimates (PREDICT), CLBCR scores (BOADICEA), and theatre time, the Wilcoxon signed-rank test was employed. Finally, the relationship between CLBCR scores (BOADICEA) and 5- and 10-year survival estimates (PREDICT) was explored using Spearman correlation analysis.

## Results

Of 272 consecutive patients identified who underwent ABR, 20 were excluded for the following reasons: 5 (1.8%) had synchronous BC, 13 (4.8%) had ductal carcinoma in situ (DCIS) only, one was male, and one was aged > 80 years, factors that precluded CLBCR estimation. After exclusions, 252 patients with a mean age of 53 years (SD 16.8) were included in the final analysis.

Table 1 illustrates the outcomes of gene testing for the entire cohort in the context of UM or BM and ABR. Of patients undergoing CPM, 14 (37.8%) had BOADICEA scores > 30%, tested gene positive, and appropriately underwent CPM and ABR. Hence, decisions for CPM were not based on genetic mutation status for the majority of patients (62.2%). Of the patients undergoing UM, only five were found to have BOADICEA scores > 30%, of whom four tested gene positive. One patient had a BOADICEA score of 30.7% and was not referred for genetic testing. Interestingly, all five of these patients at higher CLBCR were not offered CPM.

**Table 1:** Analysis of patients with genetic abnormalities undergoing unilateral and bilateral mastectomy and autologous reconstruction

Mutation status	Unilateral mastectomy	Bilateral mastectomy	Total (*N*)
No mutation/not tested	211	23	234
BRCA1 mutation	2	8	10
BRCA2 mutation	2	3	5
BRCA1 and 2 mutations	0	2	2
TP53 mutation	0	1	1
Total (*N*)	215	37	252

**Fig. 2:**
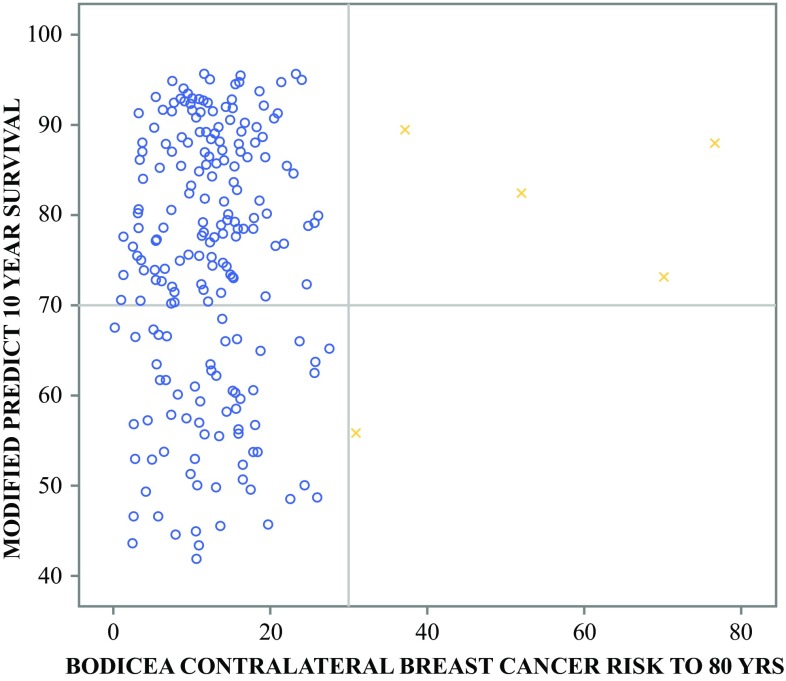
Scatter plot of BOADICEA CLBCR to 80 years (*x*-axis) verses PREDICT survival estimate at 10 years (*y*-axis) for patients receiving unilateral mastectomy. The yellow (x) represents patients at high risk of CLBC, and the blue (o) represents patients with low CLBCR. The UK NICE recommends CPM at a CLBC threshold > 30% (solid black vertical line). The figure delineates patients with a > 70% chance of surviving 10 years (solid black horizontal). *BOADICEA* Breast and Ovarian Analysis of Disease Incidence and Carrier Estimation Algorithm, *CLBCR* contralateral breast cancer risk, *CLBC* contralateral breast cancer, *NICE* National Institute for Health and Care Excellence, *CPM* contralateral prophylactic mastectomy

**Fig. 3:**
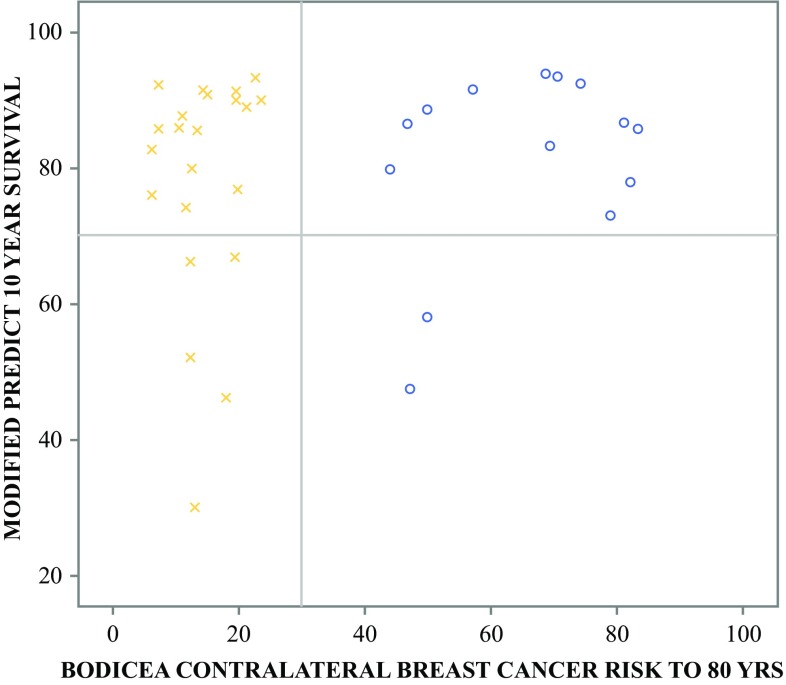
Scatter plot of BOADICEA CLBC risk to 80 years (*x*-axis) versus PREDICT survival estimate at 10 years (*y*-axis) for patients receiving bilateral mastectomy with contralateral risk-reducing mastectomy, where yellow (x) represents patients at low risk of CLBC and blue (o) represents patients with high risk of CLBC. The UK NICE recommends CPM at a CLBC threshold > 30% (solid black vertical line). The figure delineates patients with a > 70% chance of surviving 10 years (solid black horizontal). *BOADICEA* Breast and Ovarian Analysis of Disease Incidence and Carrier Estimation Algorithm, *CLBC* contralateral breast cancer, *NICE* National Institute for Health and Care Excellence, *CPM* contralateral prophylactic mastectomy

There was no correlation between BODICEA and PREDICT 10-year survival estimate scores (*p* = 0.735). As illustrated in Fig. 2, a substantial proportion of women not at high-risk of CLBC underwent CPM (20/37 [54%]). Of those who had a BODICEA score < 30%, eight (21.6%) were performed with no documented reason or at the patient’s request, five (13.5%) had contralateral non-invasive disease (DCIS, lobular carcinoma in situ [LCIS], or papilloma) with or without a FH of BC, four (10.8%) had FH, and three (8%) had ipsilateral cancer recurrence. As anticipated, the majority of patients undergoing UM had CLBCR scores < 30%. However, as seen in Fig. 3, 2.3% (5/215) of patients were identified as not undergoing CPM despite a high CLBCR score (> 30%) and ‘good’ prognosis.

The mean (SD) 5- and 10-year predicted survival for the entire cohort without any treatment was 77% (16.8%) and 60.75% (21.9%), respectively. Analysis of BOADICEA scores demonstrated the mean (SD) probability of having BRCA1 and BRCA2 mutations was 5% (19.5%) and 4% (16.1%), respectively, and the likelihood of no mutation was 91% (24.6%). Of the total 252 patients, 215 underwent UM and 37 received CPM. Table 2 highlights differences in CLBCR and survival estimates for patients undergoing unilateral versus bilateral surgery, depicting no significant difference in age (53 ± 9.6 years vs. 59 ± 9.1 years; *p* = 0.49) or 5-year (87.05 vs. 87.12; *p* = 0.21) and 10-year (75.3 vs. 79.4; *p* = 0.73) PREDICT survival estimates. The mean (SD) lifetime CLBCR in the unilateral and bilateral groups was 13.2% (15.5%) and 33.4% (26.6%), respectively. Interestingly, of patients undergoing CPM (*n* = 37), 15 (40.5%) had BRCA/P53 mutation, and 2 (5.4%) had a BOADICEA score > 30%.

**Table 2:** Comparison of clinico-pathological risk, demographic data, and outcome data between patients undergoing unilateral and bilateral surgery

Variable	Unilateral reconstruction	Bilateral reconstruction	*p* value
Mean age, years (SD)	53.5 (9.6)	50.1 (9.1)	0.493
Mean modified PREDICT 5-year survival (SD)	87.1 (10.0)	87.1 (13.0)	0.209
Mean modified PREDICT 10-year survival (SD)	75.3 (14.5)	79.4 (15.6)	0.743
Mean BOADICEA contralateral breast cancer risk to 80 years	13.2 (9.0)	33.4 (26.6)	0.000
Mean operating time, mins	451.3 (115.3)	520.8 (123.8)	0.454
Length of hospital stay, days	6.18 (2.6)	7.57 (4.4)	0.002
Complications [Clavien–Dindo] (*n*)			
0 = No complication	165	27	
1 = No intervention	1	0	
2 = Pharmacological	20	1	
3 = Surgical	28	9	
4 = Life-threatening	1	0	

*SD* standard deviation

Regarding resource implications, patients undergoing CPM had significantly increased hospital stay (mean ± SD: unilateral 6.2 ± 2.6 days vs. CPM 7.6 ± 4.4 days; *p* = 0.002). There was no significant difference in operative time [mean (SD): CPM 520.1 min (123.8) vs. unilateral 452.0 min (116.0); *p* = 0.45] (Table 2). As highlighted in Table 2, complication rates were modestly elevated in the CPM group (37% vs. 30%) and patients were twice as likely to require surgical re-intervention in the CPM group (33% vs. 17%).

## Discussion

This study demonstrates that in the absence of formal prospective CLBCR scores, a significant proportion of low-risk women undergo CPM, arguably without significant risk attenuation, and a small proportion of high-risk women are not offered CPM. Critically, a substantial proportion of patients receiving CPM have either low CLBCR and/or relatively poor prognosis from index disease, and are hence unlikely to have witnessed contralateral recurrence across their lifetime. Presently, approximately one-fifth of patients receiving CPM are classified as ‘low risk’, with a lifetime CLBCR estimate below the 30% NICE threshold. Conversely, a smaller proportion (approximately 2%) with high CLBCR and good prognosis are not receiving CPM.

This prompts the question ‘what could be driving CPM rates in patients whose estimated risk is below the CPM threshold?’ (Prior literature suggests age below 50 years,^12^ White ethnicity,^12^ higher socioeconomic status,^14^ private insurance,^14^ lobular histology,^12^ preoperative genetic testing,^14^,^15^ and MRI).^12^,^14^,^29^ In our series, low CLBCR cases justified CPM on the basis of FH or local relapse in a previously conserved breast now requiring mastectomy, most likely secondary to perceived high risk of further disease relapse. Decisions made during periods of heightened anxiety associated with initial cancer treatment lead to inflation of perceived risk and fear of recurrence.^9^,^30^,^31^ Our belief, supported by current evidence, is that CLBCR is poorly estimated and/or crudely calculated (e.g. 0.7% per annum), and we would argue in favor of objective risk scoring to augment clinical decision making, and better counsel patients considering CPM based on risk. The challenge is to conduct complex risk estimates ‘on the fly’ since computations using BOADICEA and PREDICT are not trivial. Indeed, here each BOADCIEA computation required 25–35 min, and hence the entire analysis took approximately 160 h. Therefore, an expedited surrogate calculation of CLBCR incorporating age, biology of the index BC, and FH to best approximate BOADICEA risk estimates is required. Similarly, survival estimates (PREDICT) are conducted postoperatively, and yet, for improved decision making, it would be valuable to use preoperative data to predict 10-year survival. Put simply, further work is required to consider how surrogate estimates of survival and CLBCR could be utilized prospectively to improve CPM decisions.

While, in our view, CLBCR should be the primary driver for CPM, we accept, as per both US^7^ and UK guidelines,^6^ that certain circumstances may justify CPM with CLBCR; for example, patients in whom contralateral surveillance is challenging, such as those with extremely dense breasts, and recall fatigue due to multiple further core biopsies, so called ‘screen cripples’. Arguably, more controversial are grounds for reconstructive symmetry and alleviation of psychological morbidity in risk-averse patients. Indeed, DIEP flaps may be viewed as a ‘one-off opportunity’ to achieve immediate reconstructive symmetry. A survey carried out by Montgomery and colleagues^32^ identified that the most frequent reasons for CPM is achieving symmetry.^32^ This, coupled with improvements in reliability and access to reconstructive microsurgery, may contribute to increased CPM uptake.^16^,^17^ However, in the context of increased hospital stay and psychological morbidity demonstrated in this study and by others,^5^,^19^,^20^ it is challenging to accept reconstructive symmetry as acceptable grounds for CPM in low-risk patients. Treating anxiety, depression, or fear of relapse with surgery is equally controversial, especially given the postoperative psychological morbidity disappointment and regret experienced by some women following CPM.^8^,^21^,^32^ Critically, we failed to identify patients, albeit retrospectively, in whom reconstructive symmetry or risk aversion were cited as the grounds for CPM, and yet it is challenging to conceive how these factors did not contribute to decision making in low-risk patients.

The implications of CPM decisions are being felt both in human and economic terms. On average, patients undergoing CPM stay 1.5 more days in hospital, are at increased risk of perioperative complications, and are twice as likely to require further re-intervention than patients undergoing UM and reconstruction. The increased risks of physical morbidity associated with bilateral surgery are not unique to our series. Indeed, others have observed that CPM doubles the risk.^7^,^33^,^34^ For example, Miller et al.^33^ observed that CPM patients were 2.7 times more likely to experience a major complication (odds ratio [OR] 2.66, 95% confidence interval 1.37–5.19; *p* = 0.004).^33^ Similarly, Silva et al.^34^ found that CPM was associated with increased rates of reoperation (OR 1.15, *p* = 0.029) and wound disruption (OR 2.51, *p* = 0.015).^34^ Notwithstanding the physical morbidity of surgery, many patients who undergo CPM experience regret, dissatisfaction, and disappointment with the results of surgery.^32^,^35^

Finally, compared with UM reconstruction, simultaneous CPM reconstruction has significant resource implications. Patients undergoing BM required, on average, 68 min of additional operating room (OR) time. This is valuable OR time that could have been released to operate on BCs. Moreover, in our institution, bilateral cases require two teams of attending physicians in breast oncologic surgery and plastic and reconstructive surgery, with impact on outpatient clinics and ward services. Finally, the UK financial reimbursement schedule fails to reflect the additional workload. In the National Health Service (NHS), the Health Resource Group (HRG) code for UM and ABR (HRG code = JA36Z, = £10,627, = $15,141),^36^,^37^ which compares unfavorably to BM (HRG code = JA37Z, = £13,083, $18,639).^37^ Ideally, additional resources required to reduce risk through CPM should be traded off against the longitudinal costs saved in eliminating surveillance, and indeed treating CLBC downstream.^36^

### Limitations

This study suffers limitations inherent to retrospective studies, such as recall bias and challenges surrounding prognostication and contralateral risk models. PREDICT estimates survival based on the average comorbidity for women with BC of a similar age rather than the individuals’ comorbidities, and may overestimate survival.^38^ Furthermore, our institution does not perform Ki67 measurements, which more accurately predict chemotherapy response.^36^ Extrapolation of current findings to clinical practice is challenging since PREDICT can only be calculated postoperatively following histological examination of the breast. The BOADICEA statistical tool does not account for non-familial risk factors such as BMI, reproductive factors, breast density, high-risk lesions and previous exposure to radiation,^38^ and cannot be used to guide decision making in patients with DCIS.

## Conclusions

Calculations of CLBCR and 10-year survival estimates exposes a substantial proportion of patients who are not at high-risk of contralateral cancer but in whom CPM is performed. CPM in low-risk patients is difficult to justify considering the physical morbidity, negligible survival advantage, and rationing of decisions in the current austere climate of the NHS.

## References

[1] McPherson K, Steel CM, Dixon JM (2000). Breast cancer—epidemiology, risk factors, and genetics. BMJ.

[2] Early Breast Cancer Trialists’ Collaborative Group (EBCTCG) (2005). Effects of chemotherapy and hormonal therapy for early breast cancer on recurrence and 15-year survival: an overview of the randomized trials. Lancet.

[3] Bertelsen L, Bernstein L, Olsen JH (2008). Effect of systemic adjuvant treatment on risk for contralateral breast cancer in the Women’s Environment, Cancer and Radiation Epidemiology Study. J Natl Cancer Inst.

[4] Metcalfe K, Lynch HT, Ghadirian P (2004). Contralateral breast cancer in BRCA1 and BRCA2 mutation carriers. J Clin Oncol.

[5] Lostumbo L, Carbine NE, Wallace J. Prophylactic mastectomy for the prevention of breast cancer. *Cochrane Database Syst Rev* 2010;(11):CD002748. 10.1002/14651858.CD002748.pub3 21069671

[6] National Insitute for Health and Care Excellence. Classification and care of people at risk of familial breast cancer and management of breast cancer and related risks in people with a family history of breast cancer. http://www.nice.org.uk/guidance/cg164/resources/cg164-familial-breast-cancer-full-guideline3. Accessed 7 Sept 2014.

[7] Boughey JC, Attai DJ, Chen SL (2016). Contralateral prophylactic mastectomy (CPM) consensus statement from the American Society of Breast Surgeons: data on CPM outcomes and risks. Ann Surg Oncol.

[8] Roberts A, Habibi M, Frick KD (2014). Cost-effectiveness of contralateral prophylactic mastectomy for prevention of contralateral breast cancer. Ann Surg Oncol.

[9] Wong SM, Freedman RA (2017). Growing use of contralateral prophylactic mastectomy despite no improvement in long-term survival for invasive breast cancer. Ann Surg.

[10] Neuburger J, MacNeill F, Jeevan R (2013). Trends in the use of bilateral mastectomy in England from 2002 to 2011: retrospective analysis of hospital episode statistics. BMJ Open.

[11] Rosenberg SM, Tracey MS, Meyer ME (2013). Perceptions, knowledge, and satisfaction with contralateral prophylactic mastectomy among young women with breast cancer: a cross-sectional survey. Ann Intern Med.

[12] Yi M, Hunt KK, Arun BK (2010). Factors affecting the decision of breast cancer patients to undergo contralateral prophylactic mastectomy. Cancer Prev Res.

[13] Bedrosian I, Mick R, Orel SG (2003). Changes in the surgical management of patients with breast carcinoma based on preoperative magnetic resonance imaging. Cancer.

[14] Hawley ST, Reshma J, Morrow M (2014). Social and clinical determinants of contralateral prophylactic mastectomy. JAMA Surg.

[15] Brewster AM, Parker PA (2011). Current knowledge on contralateral prophylactic mastectomy among women with sporadic breast cancer. Oncologist.

[16] Pinell-White XA, Kolegraff K, Carlson GW (2014). Predictors of contralateral prophylactic mastectomy and the impact on breast reconstruction. Ann Plast Surg.

[17] Agarwal S, Kidwell KM, Kraft CT (2015). Defining the relationship between patient decisions to undergo breast reconstruction and contralateral prophylactic mastectomy. Plast Reconstr Surg.

[18] Candido Dos Reis FJ, Wishart GC, Dicks EM (2017). An updated PREDICT breast cancer prognostication and treatment benefit prediction model with independent validation. Breast Cancer Res.

[19] Jeevan R, Cromwell DA, Brwne JP (2010). Third annual report of the National Mastectomy and Breast Reconstruction Audit.

[20] Frost MH, Hoskin TL, Hartmann LT (2011). Contralateral prophylactic mastectomy: long-term consistency of satisfaction and adverse effects and the significance of informed decision-making, quality of life, and personality traits. Ann Surg Oncol.

[21] Roberts A, Habibi M, Frick KD (2014). Cost-effectiveness of contralateral prophylactic mastectomy for prevention of contralateral breast cancer. Ann Surg Oncol.

[22] Arrington Amanda K., Jarosek Stephanie L., Virnig Beth A., Habermann Elizabeth B., Tuttle Todd M. (2009). Patient and Surgeon Characteristics Associated with Increased Use of Contralateral Prophylactic Mastectomy in Patients with Breast Cancer. Annals of Surgical Oncology.

[23] Antoniou A, Hardy R, Walker L (2008). Predicting the likelihood of carrying a BRCA1 or BRCA2 mutation: validation of BOADICEA. BRCAPRO, IBIS, Myriad and the Manchester scoring system using data from UK genetics clinics. J Med Genet.

[24] Easton D, Pharoah P, Dunning A et al. Computer programme, breast and ovarian analysis of disease incidence and carrier estimation algorithm (BOADICEA, BWA v3).

[25] Herrinton LJ, Barlow WE, Yu O (2005). Efficacy of prophylactic mastectomy in women with unilateral breast cancer: a cancer research network project. J Clin Oncol.

[26] Chung A, Huynh K, Lawrence C (2012). Ann Surg Oncol.

[27] Dindo D, Cuesta M, Bonjer H (2014). The Clavien-Dindo Classification of Surgical Complications. Treatment of postoperative complications after digestive surgery.

[28] Tracy MS, Rosenberg SM, Dominici L (2013). Breast Cancer Res Treat.

[29] Borzekowski D, Guan Y, Smith KC (2014). The Angelina effect: immediate reach, grasp, and impact of going public. Genet Med.

[30] Hawley ST, Jagsi R, Morrow M (2014). Social and clinical determinants of contralateral prophylactic mastectomy. JAMA Surg.

[31] Pesce CE, Liederbach E, Czechura T, Winchester DJ, Yao K (2014). Changing surgical trends in young patients with early stage breast cancer, 2003 to 2010: a report from the National Cancer Data Base. J Am Coll Surg.

[32] Montgomery LL, Tran KN, Heelan MC (1999). Issues of regret in women with contralateral prophylactic mastectomies. Ann Surg Oncol.

[33] Miller ME, Czechura T, Martz B, Hall ME, Pesce C, Jaskowiak N (2013). Operative risks associated with contralateral prophylactic mastectomy: a single institution experience. Ann Surg Oncol.

[34] Silva AK, Lapin B, Yao KA, Song DH, Sisco M (2015). The effect of contralateral prophylactic mastectomy on perioperative complications in women undergoing immediate breast reconstruction: a NSQIP analysis. Ann Surg Oncol.

[35] Braude L, Kirsten L, Gilchrist J, Juraskova I (2017). A systematic review of women’s satisfaction and regret following risk-reducing mastectomy. Patient Educ. Counsel..

[36] Leff DR, Ho C, Thomas H, Daniels R, Side L, Lambert F (2015). A multidisciplinary team approach minimises prophylactic mastectomy rates. Eur J Surg Oncol.

[37] NHS reference costs 2015 to 2016: information on how NHS providers spent money to provide healthcare to patients. https://www.gov.uk/government/publications/nhs-reference-costs-2015-to-2016. Accessed 16 April 2018.

[38] Wishart GC, Rakha E, Green A (2014). Inclusion of KI67 significantly improves performance of the PREDICT prognostication and prediction model for early breast cancer. BMC Cancer.

